# SUVref: reducing reconstruction-dependent variation in PET SUV

**DOI:** 10.1186/2191-219X-1-16

**Published:** 2011-08-18

**Authors:** Matthew D Kelly, Jerome M Declerck

**Affiliations:** 1 Siemens plc, Healthcare Sector, Molecular Imaging, 23/38 Hythe Bridge Street, Oxford, OX1 2EP, UK

**Keywords:** PET, SUV, reconstruction, FDG, PERCIST

## Abstract

**Background:**

We propose a new methodology, reference Standardised Uptake Value (SUV_ref_), for reducing the quantitative variation resulting from differences in reconstruction protocol. Such variation that is not directly addressed by the use of SUV or the recently proposed PERCIST can impede comparability between positron emission tomography (PET)/CT scans.

**Methods:**

SUV_ref _applies a reconstruction-protocol-specific phantom-optimised filter to clinical PET scans for the purpose of improving comparability of quantification. The ability of this filter to reduce variability due to differences in reconstruction protocol was assessed using both phantom and clinical data.

**Results:**

SUV_ref _reduced the variability between recovery coefficients measured with the NEMA image quality phantom across a range of reconstruction protocols to below that measured for a single reconstruction protocol. In addition, it enabled quantitative conformance to the recently proposed EANM guidelines. For the clinical data, a significant reduction in bias and variance in the distribution of differences in SUV, resulting from differences in reconstruction protocol, greatly reduced the number of hot spots that would be misclassified as undergoing a clinically significant change in SUV.

**Conclusions:**

SUV_ref _significantly reduces reconstruction-dependent variation in SUV measurements, enabling increased confidence in quantitative comparison of clinical images for monitoring treatment response or disease progression. This new methodology could be similarly applied to reduce variability from scanner hardware.

## Background

The Standardised Uptake Value (SUV) is a widely used metric for quantifying radiotracer (particularly ^18 ^F-2-fluoro-2-deoxy-D-glucose) uptake in clinical positron emission tomography (PET) scans. Its use is intended to provide normalisation for differences in patient size and body composition along with the dose of radiotracer injected, thereby enabling inter-study comparison between and within individual patients [1,2].

While variations in body composition and injected dose represent one significant source of variation, differences in scanner hardware and reconstruction represent another; however, these differences are not addressed by the use of SUV. These unaddressed sources of variation impede wider acceptance of PET as a quantitative imaging tool for lesion characterization, prognostic stratification and treatment monitoring, since differences in scanner hardware and reconstruction can significantly impact generated SUV [3].

A variety of proposals have been suggested to address the issue of scanner hardware/reconstruction-dependent variation in SUV. For example, the European Association of Nuclear Medicine (EANM) procedure guidelines [4], following on from the Netherlands protocol [5], provide specifications for activity concentration recovery coefficients (RC), as measured with the National Electrical Manufacturers Association (NEMA) Image Quality phantom [6]. RCs measure the ability of an imaging system to recover the true activity concentration ratio between regions filled with different activity concentrations. They are a useful indicator of clinical scanner performance, incorporating the effects of scanner resolution, sensitivity, accuracy of the various corrections performed along with the reconstruction parameters used (e.g. number of iterations and subsets, post-filter smoothing). Given these specifications, reconstruction settings should be determined for each scanner so as to generate RCs within the specified bounds. A similar approach has also been proposed by Weber and colleagues [7]. While following such an approach will reduce the variation in SUV due to differences in scanner performances and reconstruction protocol, it can negate the benefits of advances in technology which improves image quality if reconstructions are constrained to produce RCs in line with those achievable using older models of scanner. Typically, the most sensitive and advanced scanners and reconstruction techniques produce RCs which exceed the upper bounds of the protocol. Conversely, RCs that fall below the lower bounds may be improved through modification of the reconstruction parameters; however, achieving this typically requires additional iterations or reduced post-filtering, both of which increase image noise.

A different approach is used by Joshi and colleagues [8] as part of the Alzheimer's Disease Neuroimaging Initiative project. The authors apply an additional scanner-specific smoothing kernel to data from each scanner in a multi-centre trial in order to smooth all images to a common resolution. While this method succeeds in reducing the variability between datasets by 15% to 20%, it again produces images smoothed to that of the lowest resolution scanner. Furthermore, the requirement to register the clinical dataset to smoothed versions of the digital Hoffman brain phantom to determine the appropriate smoothing kernel using a voxel-wise comparison, makes the method difficult to extend to whole body data.

We propose another approach that combines reducing the variation in SUV due to differences in scanner performances and reconstruction protocol while avoiding the need to constrain reconstructions to produce RCs in line with those achievable using older models of scanner, which may negatively affect lesion detectability. The reference SUV (SUV_ref_) methodology allows users to continue to take advantage of improvements in image quality, from developments in scanner hardware and reconstruction technologies, when reviewing the clinical images. This method is not meant to address other sources of inter-scan variation in SUV, which are of biological nature. These can only be minimised by careful preparation of the patient for each scan. The aim of the SUV_ref _methodology is to reduce to a minimum the non-biological effects which may affect the calculation of SUV. The methodology can be applied to the comparison of two acquisition/reconstruction protocols as well as for multi-acquisition/reconstruction protocol comparisons. This has relevance for clinical scenarios in which an absolute SUV threshold is used to indicate malignancy, estimate prognosis or predict response to therapy. It is also applicable for centres in which a patient receives follow-up scans on a different scanner or using a different reconstruction, for example, following a scanner upgrade or in sites with multiple scanners.

## Methods

### SUVref methodology

Similar to the method described by Joshi and colleagues [8], a scanner- and reconstruction-specific smoothing filter is applied to clinical data; however, this filtered image is used only for quantification with the originally reconstructed image used for visualisation. As such, the reading physician can take advantage of the improvements in image quality and lesion detectability associated with advances in scanner hardware and reconstruction [9].

Since the filtered image is used only for quantification, filter selection is performed so as to minimise the variation in activity concentration RCs between images. For each reconstruction protocol, RCs are measured using the NEMA Image Quality (IQ) phantom, prepared and imaged as per the NEMA Standards Publication NU 2-2007 [6]. In contrast to the Standard however, the RC for each hot sphere (i.e. those with diameters 10, 13, 17 and 22 mm) is measured using the voxel with the maximum activity from a 3D volume of interest corresponding to the dimensions of the sphere. The value of the maximum voxel rather than the mean within the sphere dimensions is used to reflect the typical clinical practice for evaluation of lesions. Background activity is measured as per the NEMA Standard.

These RCs are then compared to a set of reference RCs and the root mean squared error (RMSE) calculated. This comparison is repeated following convolution of the original image with a Gaussian kernel of increasing full width half max (FWHM). The kernel size that minimises the RMSE when compared to the reference RCs is selected as the SUV_ref _filter for that scanner/reconstruction protocol combination.

The reference RCs could be determined from a specific set of scanner/reconstruction combinations used as part of a clinical trial (i.e. by taking the lowest set of RCs from the scanner/reconstruction combination with the lowest resolution). Alternatively, they could be taken from a published standard such as that defined by Boellaard et al. [4]. For this study, we have used the reference RCs published by Boellaard et al. [4]; although as the phantom was filled according to the NEMA Standards Publication NU 2-2007 [6], we have only used the RCs from the four smallest spheres. This does not affect the generality of the approach, and the method and results obtained for four spheres could be easily extended to six sphere phantoms. In addition, the reference RCs published by Boellaard et al. [4] were generated using a phantom prepared with a sphere-to-background ratio of 8:1 in contrast to the 4:1 phantom used in this study. However, this difference does not preclude the use of these published RCs as an example reference set.

### Phantom data study

The impact of SUV_ref _on variation in quantification due to differences in reconstruction was investigated using both phantom and clinical data. For the phantom studies, a ^68^Ge-filled NEMA IQ phantom, with a total activity of 116.37 MBq and a hot sphere-to-background ratio of 4:1, was acquired 15 times with a frame duration of 9 min each on a 3-ring Biograph mCT with 64-slice computed tomography (CT) and 4 × 4 mm lutetium oxyorthosilicate crystals (Siemens Healthcare, Molecular Imaging). Each of the 15 acquisitions was reconstructed with four different reconstruction protocols: OSEM 3D with 2 iterations, 24 subsets and a 5-mm FWHM Gaussian post-filter (OSEM); a point spread function reconstruction [10] with 3 iterations, 24 subsets and a 4-mm FWHM Gaussian post-filter (PSF); PSF with time of flight (TOF) with 2 iterations, 21 subsets and a 2-mm FWHM Gaussian post-filter (TOF1); and PSF-TOF with 3 iterations, 21 subsets and an all-pass filter (TOF2). All reconstructions were performed on a 200 × 200 matrix. The first three protocols are as recommended by Siemens Healthcare for whole body PET/CT scan oncological reading. The additional PSF-TOF protocol with an extra iteration was selected to provide higher RCs.

For each reconstructed dataset, the RCs were calculated, based on the maximum voxel intensity in each hot sphere. The variation in these RCs across the 15 repeats for each reconstruction protocol was measured, along with the variation between the different reconstruction protocols, using the relative standard deviation (RSD). These measurements were repeated following application of the appropriate SUV_ref _filter to each of the datasets prior to measurement of the maximum voxel intensity in each hot sphere. An SUV_ref _filter was computed for each individual dataset, and the mean filter size across all repeats for a given reconstruction protocol applied to those datasets for the analysis.

The same analysis was performed using the SUV_peak _measure as described by Wahl and colleagues [1] in the PET Response Criteria in Solid Tumors (PERCIST). PERCIST provides a structured framework for quantitative clinical reporting, with precise recommendations for how uptake in a lesion should be quantified (i.e. lean body mass corrected SUV_peak_). This builds on more general guidelines such as those published by the European Organisation for Research and Treatment of Cancer (EORTC) [11]. SUV_peak _is the mean value within a 1 cm^3 ^spherical region positioned within a lesion so as to maximise this value. The motivation behind SUV_peak _was to provide a value less sensitive to noise than the SUV_max _and less dependent on lesion delineation than SUV_mean_. Although not intended to address reconstruction and scanner-dependent variation, it also involves the application of a smoothing filter (although non-Gaussian) to an image for the purpose of quantification, which combined with its potential acceptance by the PET community makes it an interesting measure for comparison with the SUV_ref _methodology.

Finally, a combination of SUV_ref _and SUV_peak _was evaluated, SUV_ref,peak _in which the peak value is computed from the SUV_ref _filtered image.

### Clinical data study

For the clinical data, sinograms and attenuation CTs were collected for ten oncology patients with a variety of malignancies acquired and reconstructed using the same scanner and four reconstruction protocols used in the phantom study (data courtesy of Lemmen-Holton PETCT, Grand Rapids, MI). The mean patient dose was 446 MBq (SD, 66 MBq). For each patient, 50 hotspots (i.e. local maxima) corresponding to malignant and normal physiological uptake were manually delineated and the SUV_max _measured for each of the 4 reconstructions. The mean SUV_max _and volume for the selected hotspots were 4.8 (SD, 4.9) and 13.1 cm^3 ^(SD, 21.6 cm^3^), respectively. The volume reported was that enclosed within an isocontour corresponding to 40% of the SUV_max_. The change in SUV_max _for each hotspot across each possible pairing of the four reconstructions was then calculated. Any change in SUV_max _therefore reflected the effect of differences in reconstruction protocol alone since the underlying sinogram data was the same for each comparison. Specifically, the percentage change in SUV_max _(*Δ*_SUVmax_) was calculated as follows:

(1) $$\Delta_{\text{SUV}\max}=\frac{\left\|{\text{SUV}}_{a}-{\text{SUV}}_{b}\right\|}{\left({\text{SUV}}_{a}+{\text{SUV}}_{b}\right)/2}\times 100$$

where SUV*_a _*is the SUV_max _measured for a given hotspot on the image reconstructed with protocol *a*, and SUV*_b _*is the SUV_max _measured for the corresponding hotspot on the image reconstructed with protocol *b*. Reconstruction protocols *a *and *b *represent one of the six possible pairings of the four reconstruction protocols used. For each pairing, the reconstruction with the largest SUV_ref _filter computed in the phantom study was selected as protocol *a*.

This analysis was repeated using the same set of 500 hotspots, following application of the appropriate SUV_ref _filter to each reconstruction prior to measurement of the maximum voxel intensity, to compute percentage change in SUV_ref _(*Δ*_SUVref_). The SUV_ref _filters used were those derived from the ^68^Ge phantom study described above. The same analysis was also repeated using the SUV_peak _measure to compute *Δ*_SUVpeak_.

The sensitivity of the SUV_ref _methodology to filter size was assessed by applying non-optimal SUV_ref _filters and measuring the effect on *Δ*_SUVref_. This assessment was performed for the comparison of PSF with OSEM and for TOF1 with OSEM. The non-optimal filters for each pairwise comparison were selected by increasing the FWHM of the mean SUV_ref _filter for the reconstruction with the lowest RCs (i.e. OSEM) by twice the standard deviation (SD) of the mean filter FWHM for that reconstruction from the phantom study, and decreasing the FWHM of the optimal filter for the reconstruction with the highest RCs (i.e. PSF or TOF1) by the corresponding amount.

The effect of hotspot location on the performance of SUV_ref _was assessed by separating the set of 500 clinical hotspots into two groups, lateral and medial. The threshold for this separation was arbitrarily selected as 75 mm from the centre of the transaxial field of view since this resulted in equal size groups. The motivation for this comparison was to evaluate any effect on SUV_ref _performance of comparing PSF-based reconstructions with an improved resolution uniformity throughout the transaxial FOV, compared with a traditional OSEM reconstruction [10].

Finally, to investigate the impact of SUV_ref _on measuring response, a subset of 25 lung hotspots were extracted from the original 500 clinical hotspots. All 300 possible pairwise combinations of these hotspots were then used to simulate response studies, with one of each pair providing the baseline measurement and the other the follow-up measurement. For each simulated response study, the percentage change was calculated using both SUV_max _and SUV_ref_, as described above, for each of the four reconstruction protocols, with the same reconstruction protocol used per simulated measurement of response. The mean absolute difference in calculated percentage change for each pair of hotspots across the four reconstruction protocols was then compared for SUV_max _and SUV_ref_.

## Results

### Phantom data study

The SUV_ref _filters computed for the four reconstruction protocols, in order to minimise the difference in RCs when compared to the reference values published by Boellaard et al. [4], are shown in Table 1. The data reconstructed with OSEM required the smallest additional filter (3.3-mm FWHM), while the TOF2 data with the additional iteration required the largest (7.1-mm FWHM). This was as expected given the contrast to noise improvements observed in images reconstructed with the PSF and PSF-TOF reconstruction algorithms [12].

**Table 1:** Mean SUV_ref _filters computed for the four reconstruction protocols

**Reconstruction protocol**^**a**^	SUVref filter FWHM (mm)
OSEM 2i24s5 mm (OSEM)	3.3 (0.54)
PSF 3i24s4 mm (PSF)	6.5 (0.21)
PSF-TOF 2i21s2 mm (TOF1)	6.7 (0.29)
PSF-TOF 3i21s0 mm (TOF2)	7.1 (0.28)

Mean (with standard deviation in parenthesis). ^a^i, number of iterations; s, number of subsets; mm, FWHM in millimeters of Gaussian post-reconstruction filter.

**Figure 1:**
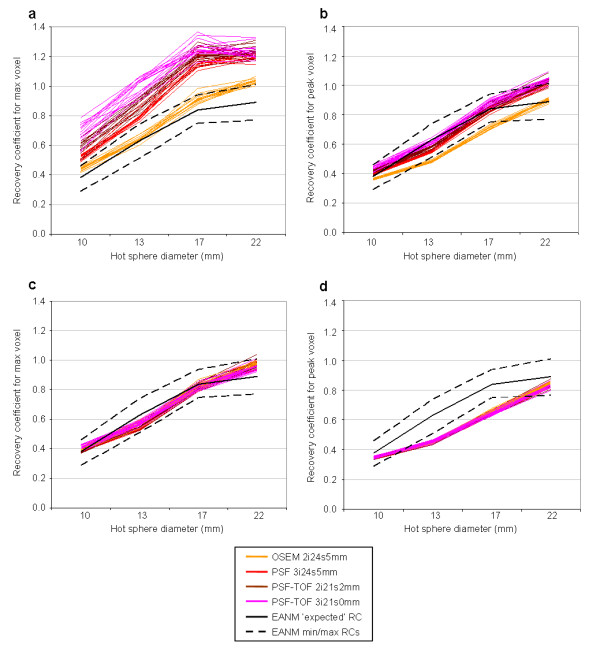
**Plots of RCs measured for the 15 repeats with each of the 4 reconstructions protocols**. Using (**a**) SUV_max_, (**b**) SUV_peak_, (**c**) SUV_ref _and (**d**) SUV_ref,peak _with the reconstruction-specific filters applied. The solid- and dashed-black lines show the expected and min/max RCs, respectively, as reported in the EANM procedure guidelines [4].

The effect of applying these SUV_ref _filters on the RCs measured for the phantom studies is shown in Figure 1. Figure 1a shows the RCs measured using the max voxel value in the original data. All reconstruction protocols with the exception of OSEM fall entirely outside the EANM specifications [4] (denoted by the dashed lines), and all but one of these OSEM reconstructions have at least one RC above the proposed maximum specification. Figure 1c shows the RCs measured following application of the SUV_ref _filter. With the exception of the 22-mm sphere in 2 of the 60 reconstructed repeats, all points lie within the bounds defined in the EANM specification [4]. Although the EANM bounds are for the maximum voxel value, the RCs for SUV_peak _(Figure 1b) and SUV_ref,peak _(Figure 1d) are also shown. For SUV_peak_, 55 of the 60 reconstruction repeats have at least one RC either above or below the EANM-specified bounds, with all repeats having at least one point outside the bounds for SUV_ref,peak_. It is also worth noting that with SUV_max_, all reconstructions produce RCs greater than 1 for at least the largest hot sphere. An RC greater than 1 is most likely due to the positive bias of selecting the maximum voxel in noisy data [13], although could also result from imperfections in the scatter correction or cross-calibration of the scanner. This will be more apparent for reconstructions with better RC and higher noise; although improvements in RC beyond a certain point will have minimal impact for larger spheres. With the additional smoothing of SUV_peak_, SUV_ref _and SUV_ref,peak_, far fewer RCs are greater than 1.

The variation within each reconstruction protocol and across all protocols is presented in Table 2. The mean RSD is significantly reduced for all intra-reconstruction comparisons simply as a result of applying a smoothing filter, as shown with both SUV_ref _and SUV_peak_. However, a significantly larger reduction in mean RSD across all protocols was seen with SUV_ref _(and SUV_ref,peak_) when compared to SUV_max _(and SUV_peak_). In fact, the mean RSD across all protocols with SUV_ref _(and SUV_ref,peak_) was smaller than the intra-reconstruction mean RSD for all but the OSEM reconstructed data with SUV_max_. This implies that with the application of an appropriate SUV_ref _filter, there is less variance in a set of data from a range of different reconstructions than within data reconstructed with the same protocol when using SUV_max_.

**Table 2:** Mean RSD of the RCs for each reconstruction protocol and across all protocols

Reconstruction protocol	**Mean RSD with SUV**_**max **_**(%)**	**Mean RSD with SUV**_**peak **_**(%)**	**Mean RSD with SUV**_**ref **_**(%)**	**Mean RSD with SUV**_**ref,peak **_**(%)**
OSEM	2.81	1.59	2.28	1.46
PSF	3.25	1.80	2.00	1.49
TOF1	4.69	2.32	2.58	1.70
TOF2	5.70	2.51	2.68	1.72
All protocols	13.60	7.75	2.85	1.72

Mean RSD of the RCs for the 15 repeats per reconstruction protocol and across all reconstruction protocols for SUV_max_, SUV_ref _and SUV_peak_. Reduction in RSD with both SUV_ref _and SUV_peak _for all intra-reconstruction protocol comparisons, in addition to across all protocols, was significant (*P *< 0.01 with paired two-tailed Student's *t*-test).

### Clinical data study

**Figure 2:**
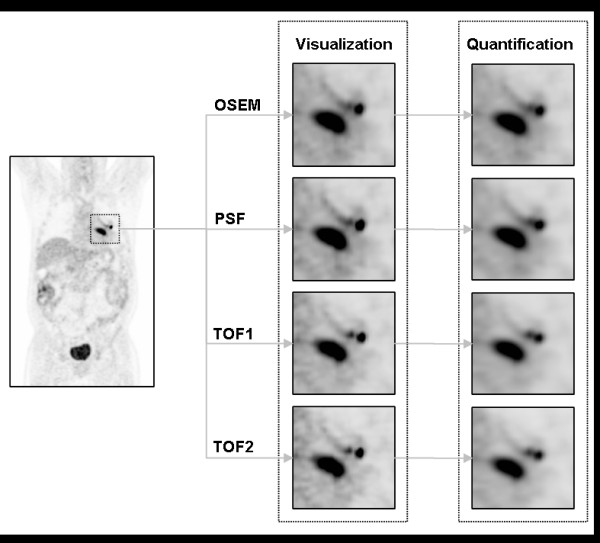
**Coronal slice through one of the clinical datasets**. The slice demonstrating the progressive improvement in visual image quality with increasingly advanced reconstruction protocols. A visual indication of the effect of applying the SUV_ref _filter to the image volumes is also shown, even if that filtered image is not used for reading.

**Figure 3:**
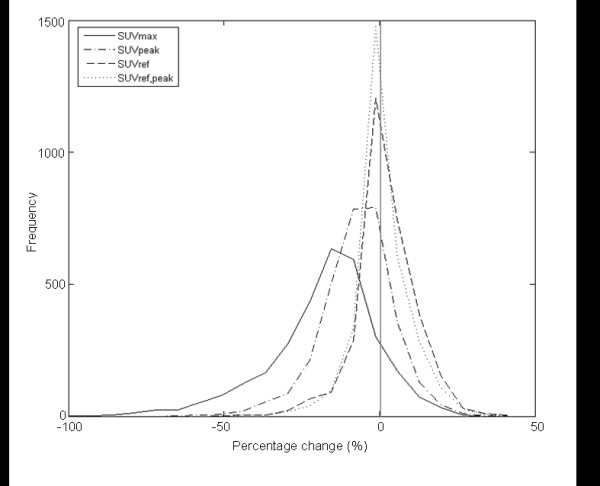
**Distribution of *Δ*_SUVmax_, *Δ*_SUVpeak_, *Δ*_SUVref _and *Δ*_SUVref. peak _for the clinical datasets**. *Δ*_SUVmax _(solid line), *Δ*_SUVpeak _(dash-dot line), *Δ*_SUVref _(dashed line) and *Δ*_SUVref.peak _(dotted line). The mean (and SD) for SUV_max _was -17.8% (17.4), for SUV_peak _-7.19% (11.56), for SUV_ref _-1.98% (9.42) and for SUV_ref,peak _-0.84% (8.61). The difference between each distribution is significant (*P *< 0.001 with paired two-tailed Student's *t *test).

For the clinical data, the same four reconstruction protocols were used and the SUV_ref _filter sizes computed with the corresponding phantom studies applied (Figure 2). Figure 3 shows the distribution in percentage changes for *Δ*_SUVmax_, Δ_SUVref_, *Δ*_SUVpeak _and *Δ*_SUVref,peak_. Both bias and variance are reduced with SUV_ref_, from -17.8% (17.4 SD) with SUV_max _to -1.98% (9.42 SD). SUV_peak _has an intermediate bias and variance of -7.19% (11.56 SD), with SUV_ref,peak _having the smallest bias and variance of 0.84% (8.61 SD).

The reduction of bias with SUV_ref _to close to zero means there is no longer a higher maximum with one reconstruction versus another. The potential clinical impact of the reduction in bias and variance with SUV_ref _can be evaluated by considering the use of a fixed threshold of percentage change in order to determine disease progression or treatment response. Table 3 shows the percentage of hotspots having a *Δ*_SUVmax_, *Δ*_SUVref_, *Δ*_SUVpeak _or *Δ*_SUVref,peak _greater than either 10%, 20% or 30%. This percentage can be considered as the proportion of hotspots that would be incorrectly classified as having a clinically relevant change despite the underlying sinogram data being identical, with any change being purely a result of differences in reconstruction protocol. In all cases, the percentage of hotspots with a percentage change above the threshold is greatly reduced with SUV_ref _with an intermediate reduction seen for SUV_peak _and the greatest reduction with SUV_ref,peak_. For example, even with a conservative PERCIST-recommended threshold of 30%, a clinically relevant change was incorrectly identified in nearly 20% of hotspots when using SUV_max_, compared to just 1% with SUV_ref_. For SUV_peak_, nearly 4% of hotspots would be incorrectly classified as undergoing a clinically significant change.

**Table 3:** Percentage of hotspots with a *Δ_SUVmax_, Δ_SUVpeak_, Δ_SUVref _*or *Δ_SUVref,peak _*greater than specified difference threshold

Difference threshold	**Percentage with SUV**_**max **_**(%)**	**Percentage with SUV**_**peak **_**(%)**	**Percentage with SUV**_**ref **_**(%)**	**Percentage with SUV**_**ref,peak **_**(%)**
10%	70.1	41.5	24.7	19.8
20%	37.6	12.3	5.7	3.7
30%	19.9	3.9	1.0	0.7

Percentage of hotspots with a *Δ_SUVmax_, Δ_SUVpeak_, Δ_SUVref _*or *Δ_SUVref,peak _*greater than the specified difference threshold across all six pairwise combinations of the four reconstruction protocols evaluated.

**Figure 4:**
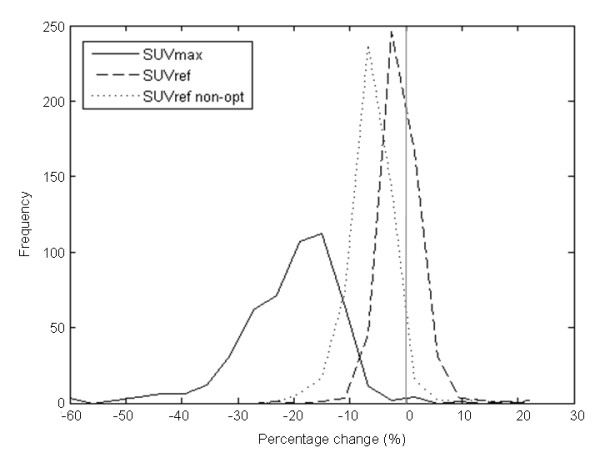
**Distribution of *Δ*_SUVmax _and *Δ*_SUVref _with non-optimal filters for PSF and OSEM reconstruction protocols**. *Δ*_SUVmax _(solid line) and *Δ*_SUVref _(dashed line). The mean (and SD) for *Δ*_SUVmax _was -20.3% (9.1) and for *Δ*_SUVref _-1.00% (3.54). Also shown with a dotted line is the distribution of *Δ*_SUVref _with the application of suboptimal filters. The mean (and SD) for this non-optimal *Δ*_SUVref _is -6.25% (3.89). The difference between each distribution is significant (*P *< 0.001 with paired two-tailed Student's *t *test).

The sensitivity of this reduction in bias and variance to filter size was investigated using non-optimal SUV_ref _filters for two reconstruction comparisons. For the first comparison, PSF versus OSEM, the change in the distribution of *Δ_SUVref _*for the non-optimal filters versus the optimal filters is shown in Figure 4 and Table 4. The non-optimal filters used, 6.1 and 4.4-mm FWHM, respectively, were both closer to one another by twice the respective SD from the mean filters identified in the phantom study (6.5 and 3.3 mm, respectively). This is aimed at simulating a "worst case scenario" in the situation where the SUV_ref _filters would not have been estimated optimally. The reduction in bias and variance, along with the reduction in number of hotspots with a percentage change above the individual thresholds, is smaller when using the non-optimal filters; however, when compared to SUV_max_, the reduction even with non-optimal filters is still significant.

**Table 4:** Effect of non-optimal filters on *Δ_SUVmax _*and *Δ_SUVref_*, for PSF and OSEM reconstruction protocols

Difference threshold	**Percentage with SUV**_**max **_**(%)**	**Percentage with SUV**_**ref **_**(%)**	**Percentage with non-optimal SUV**_**ref **_**(%)**
10%	93.2	1.4	12.6
20%	44.6	0.6	0.8
30%	12.2	0.0	0.0

Percentage of hotspots with a *Δ_SUVmax _*or *Δ_SUVref _*greater than the specified threshold for the comparison of PSF and OSEM reconstruction protocols. Values are also shown when non-optimal SUV_ref _filters are applied.

**Figure 5:**
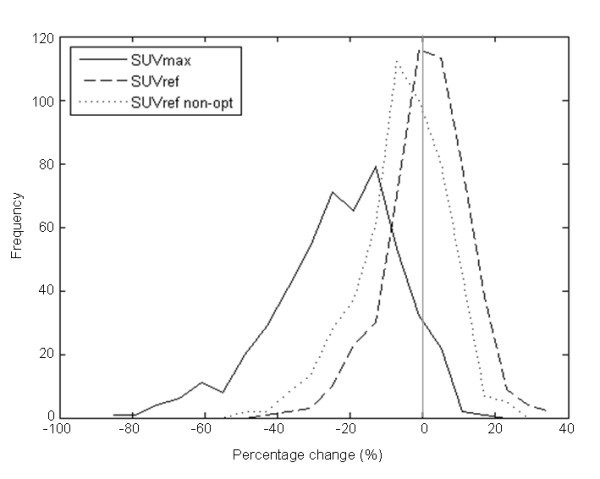
**Distribution of *Δ*_SUVmax _and *Δ*_SUVref _with non-optimal filters for TOF1 and OSEM reconstruction protocols**. *Δ*_SUVmax _(solid line) and *Δ*_SUVref _(dashed line). The mean (and SD) for *Δ*_SUVmax _was -23.4% (17.2) and for *Δ*_SUVref _1.23% (11.2). Also shown with a dotted line is the distribution of *Δ*_SUVref _with the application of suboptimal filters. The mean (and SD) for this non-optimal *Δ*_SUVref _is -5.69% (12.1). The difference between each distribution is significant (*P *< 0.001 with paired two-tailed Student's *t *test).

The same behaviour can be seen with the second comparison, TOF1 versus OSEM, Figure 5and Table 5. Again, a smaller, but still significant, reduction in bias and variance, and number of hotspots with a percentage change above the individual thresholds, is observed when non-optimal filters are used.

**Table 5:** Effect of non-optimal filters on *Δ_SUVmax _*and *Δ_SUVref_*, for TOF1 and OSEM reconstruction protocols

Difference threshold	Percentage with SUV_max _(%)	Percentage with SUV_ref _(%)	Percentage with non-optimal SUV_ref _(%)
10%	78.4	34.4	38.0
20%	53.4	8.4	13.2
30%	32.0	1.4	3.4

Percentage of hotspots with a *Δ_SUVmax _*or *Δ_SUVref _*greater than the specified threshold for the comparison of TOF1 and OSEM reconstruction protocols. Values are also shown when non-optimal SUV_ref _filters are applied.

**Figure 6:**
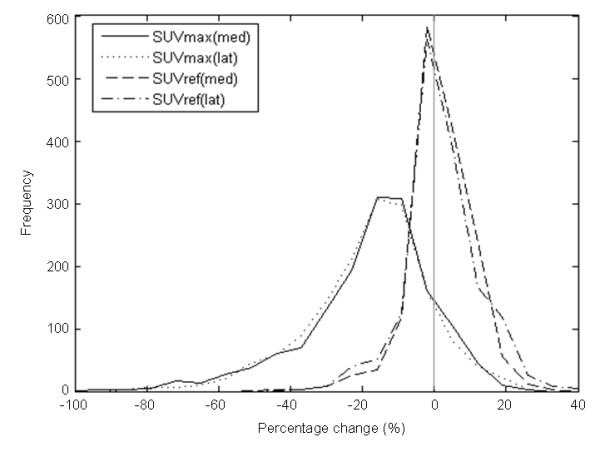
**Distribution of *Δ*_SUVmax _and *Δ*_SUVref _for medial and lateral (solid and dashed lines, respectively) hotspots**. The mean (and SD) for medial *Δ*_SUVmax _was -17.8% (17.8), for medial *Δ*_SUVref _was 1.92% (8.74), for lateral *Δ*_SUVmax _was -18.0% (17.0), for lateral *Δ*_SUVref _was 2.04% (10.1). There is no significant difference between the medial and lateral *Δ*_SUVmax _distributions (*P *= 0.72) or *Δ*_SUVref _distributions (*P *= 0.73).

The effect of hotspot distance from centre of the transaxial field of view on *Δ*_SUVref _is shown in Figure 5 and Table 6. No significant difference between lateral and medial *Δ*_SUVref _or *Δ*_SUVmax _distributions was observed (Figure 6). This is reflected in the number of hotspots with a percentage difference above the thresholds specified (Table 6).

**Table 6:** Effect of hotspot location on *Δ_SUVmax _*and *Δ_SUVref_*

Difference threshold	**Percentage with SUV**_**max **_**(%)**		**Percentage with SUV**_**ref **_**(%)**	
	Medial	Lateral	Medial	Lateral
10%	68.81	71.41	22.40	27.32
20%	37.91	37.69	4.28	7.51
30%	20.35	19.90	0.67	0.99

Percentage of medial and lateral hotspots with a *Δ_SUVmax _*or *Δ_SUVref _*greater than the specified threshold for all six pairwise combinations of the four reconstruction protocols evaluated.

Finally, the assessment of the impact of SUV_ref _on response assessment, when the same reconstruction protocol is used for both the baseline and follow-up study, showed a significant reduction in the mean absolute difference in percentage change, as measured across the four different reconstruction protocols, from 11.8% (8.7% SD) with SUV_max _to 6.8% (6.2% SD) with SUV_ref _(*P *< 0.01 with the Wilcoxon Matched-Pairs Signed-Ranks Test).

## Discussion

Variations in reconstruction protocol can have a major effect on quantifiable parameters such as contrast recovery. For example, in the phantom experiments described above, the RC for the 10-mm hot sphere varies from 0.42 to 0.78 and from 1.01 to 1.33 for the 22-mm hot sphere. Following application of the appropriate SUV_ref _filters, this variation reduces to 0.38 to 0.43 for the 10-mm hot sphere and 0.93 to 1.04 for the 22-mm hot sphere. In fact, with SUV_ref _the mean variation in RC across all reconstruction protocols studied is smaller than the mean variation in RC within a single reconstruction protocol. A reduction in RC variation was also observed with the PERCIST measure SUV_peak_; however, the variation across all reconstruction protocols was significantly larger than for SUV_ref_. The combination of SUV_ref _and SUV_peak _in SUV_ref,peak _reduces the variation across reconstruction protocols further still.

In addition to reducing the variation resulting from differences in reconstruction protocol, SUV_ref _can be defined to produce RCs within the bounds specified by the recently published EANM specification [4]. Given all reconstructions evaluated with SUV_max _produced RCs that were above the EANM-specified bounds, application of the SUV_ref _filter would ensure clinical sites using these reconstruction protocols produced quantifiably conforming values whilst allowing them to take advantage of improvements in image quality associated with advanced reconstruction protocols. With SUV_peak_, more than 90% of reconstructions evaluated produced RCs outside EANM-specified bounds. Given the distribution of these outliers both above and below the specified bounds, significant widening of the bounds would be required to accommodate SUV_peak_, and therefore reduce the benefit of the specification.

The potential clinical impact of the reductions in RC variability with SUV_ref _was presented in Table 3. For example, if a percentage change in SUV_max _of greater than 30% is selected as signifying a clinically relevant change in the status of a lesion, either disease progression or treatment response, then for the combination of reconstruction protocols evaluated, a clinically relevant change would be incorrectly observed nearly 20% of the time, compared to just 1% with SUV_ref_, when in fact there is no change in the underlying data. This reduction results from the reduction in bias and variation shown in Figure 2. In PERCIST, a threshold of 30% is used with SUV_peak _to signify either metabolic disease progression or treatment response [1]. With the combination of reconstruction protocols evaluated in this study, a hotspot would be incorrectly classified nearly 4% of the time.

The use of such a conservative threshold (i.e. 30%) is a consequence of the intrinsic variability in repeat PET scans, biological variability and the need to account for inter-scanner variability and aims to reduce the number of incorrectly classified responders, albeit at the cost of sensitivity. The adoption of a methodology such as SUV_ref _may enable the use of a less conservative threshold, by reducing the need to accommodate for inter-scanner variability, thus increasing sensitivity without increasing the number of incorrectly classified responders.

The combination of SUV_ref _and SUV_peak _in SUV_ref,peak _results in a further reduction in the percentage of incorrectly classified lesions (0.7%). This is due to the additional smoothing inherent in the calculation of the peak value.

The sensitivity of the SUV_ref _methodology to SUV_ref _filter size was investigated using non-optimal filters. In both reconstruction protocol comparisons (PSF versus OSEM and TOF1 versus OSEM), the application of non-optimal filters reduced the improvement in quantitative comparability provided by the optimal SUV_ref _filters as would be expected. Despite this, the improvement when compared to SUV_max _was still significant. Given the non-optimal filter, sizes were used each 2 SDs closer together than the optimal filter sizes, the chance of such suboptimal filters being selected by chance is very small, particularly if multiple phantom acquisitions are performed for filter selection (for instance, three repeats are recommended in the NEMA Standard [6]).

Considering the difference in resolution uniformity within the transaxial field of view with PSF-based reconstructions versus traditional OSEM, the effect of hotspot location was assessed. In the comparison of medial (< 75 mm from centre of transaxial FOV) versus lateral lesions (≥75 mm from centre of transaxial FOV), no significant difference in the distribution of percentage differences for either SUV_max _of SUV_ref _was observed.

In addition to reducing the variation in quantification of uptake for individual hotspots across different reconstruction protocols, SUV_ref _also significantly reduces the variation in assessments of change in uptake when both the baseline and follow-up scans are reconstructed using the same protocol. This in turn reduces the likelihood that the assessment of response for a given patient would differ between sites purely as a result of differences in reconstruction protocol.

While this study has evaluated the ability of SUV_ref _to reduce reconstruction-dependent variation in SUV, similar performance would be expected for scanner-dependent variation since this would also manifest mainly as a difference in RC.

It is also worth noting that an alternative solution could be to reconstruct the image with two protocols, one optimised for visual review and the other conforming to the EANM guidelines. However, the SUV_ref _methodology has the advantage of avoiding the additional burden of reconstructing, storing and reviewing a second version of every data set.

## Conclusion

SUV_ref _significantly reduces reconstruction-dependent variation in SUV measurements, while preserving the benefits of improved image quality through advances in reconstruction and scanner technology. This reduction in variation provides increased confidence in quantitative comparison of clinical images for monitoring treatment response or disease progression.

## Competing interests

This research was funded by Siemens Healthcare of which both M. Kelly and J. Declerck are employees.

## Authors' contributions

MK and JD conceived and designed the study. MK carried out the experiments, analysis and drafted the manuscript. Both authors read and approved the final manuscript.
